# Does Invasive Treatment Increase the Long-Term Survival of ST-Elevation Myocardial Infarction Patients with a History of Coronary Artery Bypass Graft Surgery?

**Published:** 2019-07

**Authors:** Mohsen Taghavi Shavazi, Seyedmohammad Saadatagah, Hassan Aghajani, Hamidreza Poorhosseini, Mojtaba Salarifar, Alireza Amirzadegan, Alimohammd Hajzeinali, Mohammad Alidoosti, Reyhaneh Aghajani, Ebrahim Neamatipour

**Affiliations:** *Tehran Heart Center, Tehran University of Medical Sciences, Tehran, Iran.*

**Keywords:** *Acute coronary syndrome*, *ST elevation myocardial infarction*, *Survival analysis*, *Thrombolytic therapy*, *Percutaneous coronary intervention*, *Coronary artery bypass*

## Abstract

**Background: **Although invasive treatments such as primary percutaneous coronary intervention (PPCI) are the treatment of choice in ST-elevation myocardial infarction (STEMI) patients, the survival benefit of this treatment in patients with a history of coronary artery bypass graft (CABG) has yet to be fully evaluated.

**Methods:** In this historical cohort study, 251 STEMI patients with a history of CABG between 2007 and 2017 were stratified into 3 groups of no reperfusion, thrombolytic, and PPCI based on their treatment strategy. Baseline clinical characteristics, details of the STEMI event, and the course of hospitalization were evaluated for all patients and they were followed up until May 2018 to assess all-cause mortality.

**Results: **The mean age of the study population was 64.019.45 years, and 81.7% of them were male. The median follow-up time was 1304 (IQR_25%-75%_: 571–2269) days, the short-term (1 month) mortality rate was 5.97%, and the long-term mortality rate was 15.1%. There was no significant difference between the 3 different strategies in terms of survival. In the fully adjusted multivariate analysis, cardiopulmonary resuscitation (HR: 15.06, 95% CI: 2.25–101.14, P=0.005) was significantly associated with short-term mortality, while diabetes (HR: 5.95, 95% CI: 2.03–17.44, P=0.001), opium abuse (HR: 4.85, 95% CI: 1.45–16.23, P=0.010), and cardiopulmonary resuscitation (HR: 11.73, 95% CI: 3.44–40.28, P=0.001) were significantly associated with long-term mortality.

**Conclusion: **Our results failed to show the superiority of invasive treatment in terms of survival. Further studies regarding the advantages and disadvantages of invasive treatment in post-CABG patients are required.

## Introduction

A History of coronary artery bypass graft (CABG) surgery in a patient who presents with a suspicious ST-elevation myocardial infarction (STEMI) poses a diagnostic and therapeutic challenge. Although nowadays invasive treatments such as primary percutaneous coronary intervention (PPCI) are deemed the treatment of choice in STEMI patients,^1^ the efficacy of such treatments in a special subgroup of patients including senile patients,^2^^, ^^3^ those with a history of CABG, ^4^^-^^7^  and those with severe renal dysfunction^   8 ^ should be evaluated carefully.

In comparison with CABG-naïve patients, post-CABG patients are older,^4^^-^^7^ exhibit a higher prevalence of cardiac risk factors,^4^^-^^7^ suffer from more comorbidities,^4^^-^^7^^, ^^9^ and have lower ejection fractions.^5^^-^^7^ In many landmark studies on the efficacy of reperfusion strategies in the management of STEMI, post-CABG patients were either excluded^10^^-^^12^ or comprised just a small percentage of the study population.^13^^-^^16^ Consequently, it has remained unknown whether or not the results of such studies could be extended to this group of patients. 

All previous studies have compared the outcome of PPCI in patients with and without a history of CABG, and different results have been reported.^4^^-^^7^ Some studies were in favor of higher mortality of STEMI in post-CABG patients,^4^ whereas others supported the similar outcome of STEMI in patients with and without a history of CABG.^7^ However, there has yet to be a study on the comparison between different treatment strategies in this particular group of patients. We designed the present study to compare the long-term outcome of different treatment strategies in the management of STEMI in patients with a previous history of CABG.

## Methods

This is a historical cohort study on all patients with a history of CABG who were admitted to Tehran Heart Center (THC) with a diagnosis of STEMI between 2007 and 2017 (whether or not the initial management was done at THC). The exclusion criteria were non-STEMI and ST-elevation caused by etiologies other than STEMI. Because of the more complex nature of patients with concomitant valvular surgery, patients with a history of prosthetic valve implantation along with CABG were also excluded. 

Based on their reperfusion strategy, the patients were stratified into different groups of no reperfusion (if the patient did not receive thrombolytic agents in the first 12 hours or PCI within 24 hours of symptom onset), thrombolytic group (if the pharmacologic thrombolysis was performed within 12 hours of symptom onset and no PCI was performed in the next 24 hours), PPCI (if PPCI was the initial reperfusion strategy and was performed within 24 hours of symptom onset), and rescue-facilitated PCI (if PCI was performed within 24 hours after the initiation of thrombolytic therapy). Nevertheless, since there was no patient to match the definition of the fourth group, the study population was categorized into the first 3 groups.

All the patients were followed up until May 2018 whether they were dead or alive. The date of death was considered the end point; whereas for those who survived, May 2018 was considered the end of follow-up. The entire study population provided informed consent, and the study protocol was approved by the Ethics Committee of THC and was in compliance with the principles of the Declaration of Helsinki.

All data on mortality, the length of hospital stay, risk factors, demographic features, and different therapeutic strategies over the 11-year study period were extracted from the Data Bank of THC and evaluated. Along with self-reports regarding previously diagnosed diseases necessitating medical treatment, diabetes mellitus was defined as a fasting blood glucose level >126 mg/dL and/or an HbA1C level >7% mmol/mol; hypertension was defined as being on antihypertensive drugs or having blood pressure >140/90 mmHg; and dyslipidemia was defined as a high-density lipoprotein level <40 mg/dL in men and <50 mg/dL in women, a triglyceride level >250 mg/dL, and a low-density lipoprotein level >100 mg/dL. Current cigarette smoking and also a history of cigarette smoking were categorized as smoking, and current or previous abuse of opium derivatives was defined as opium abuse. Chronic kidney disease was defined as a glomerular filtration rate <60 cc/min/kg, and acute kidney injury was defined as an increase in creatinine >50% or >0.3 mg/dL from baseline. Additionally, oliguria, defined as urine output <0.5 mL/min for >6 hours, was also considered to constitute acute kidney injury. 

The continuous variables are presented as the mean ± the standard deviations (SDs) for those with a normal distribution and as the median (interquartile ranges [IQR_25%-75%_]) for those without a normal distribution. The dichotomous variables are presented as numbers (percentages). Group comparisons for the categorical and continuous variables were performed using the ^2^, Fisher Exact, Mann–Whitney, and Kruskal–Wallis tests, as appropriate. A P value <0.05 was considered statistically significant.

Cox regression modeling was used for survival analysis, and corresponding Kaplan–Meier plots, along with log-rank P values, were presented. The variables with a P value <0.20 in the univariate analysis were included in the final multivariate Cox regression model. Adjusted hazard ratios (HRs) for all-cause mortality and 95% confidence intervals (95% CIs) are presented. The statistical analyses were performed using SPSS software, version 22 (IBM SPSS Statistics for Windows, Version 22.0. Armonk, NY: IBM Corp.).

## Results

**Figure 1:**
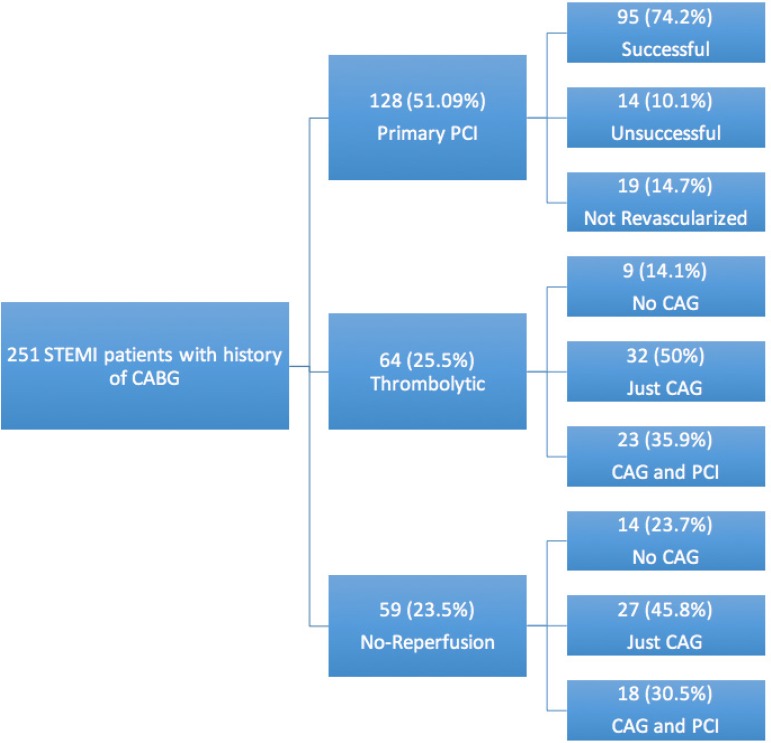
Flowchart of the performed reperfusion strategies in the study population

STEMI, ST-elevation myocardial infarction; CABG, Coronary artery bypass graft surgery; PCI, Percutaneous coronary intervention; CAG, Coronary angiography

**Figure 2:**
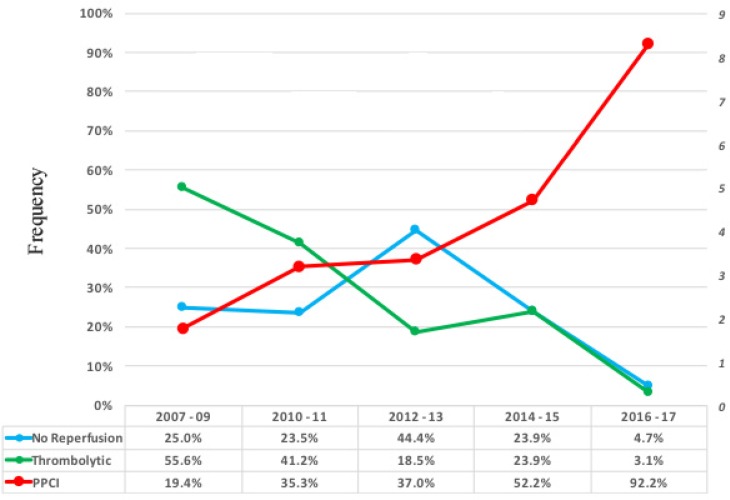
Frequency of the different reperfusion strategies based on the year of admission

The study population consisted of 251 patients at a mean age of 64.019.45 years, and men comprised 81.7% of the patients. As is shown in Figure 1, the inclusion and exclusion criteria were fulfilled by 251 patients, who were incorporated in the final analysis. There were 59 (23.5%) patients in the no-reperfusion group, 64 (25.5%) patients in the thrombolytic group, and 128 (51.0%) patients in the PPCI group. There was a rise in the number of PPCI procedures during the study period, with 92.2% of the patients undergoing the procedure between 2016 and 2017. Along with changes in the reperfusion strategy, the median of the length of hospital stay was also reduced from 8.0 (IQR_25%-75%_: 6.0–11.0) days to 5.0 (IQR_25%-75%_: 4.0–8.0) days during the period of the study (Figure 2). 

The baseline clinical status of the study population, the index event characteristics, and the indices of severity were compared between the groups (Table 1 and Table 2). Although the study population was not randomized, all 3 groups were largely similar, and no significant difference was observed between them except for the presence of hypertension (P=0.009) and the involvement of the lateral leads (P=0.006). The median of the ischemic time (onset of symptoms to reperfusion) was 3.0 hours (IQR_25%-75%_: 2.5–5.0) in the thrombolytic group and 4.0 hours (IQR_25%-75%_: 2.5-6.9) in the PPCI group (P=0.042).

The prevalence rates of diabetes mellitus, hypertension, dyslipidemia, cigarette smoking, and opium abuse were 36.3%, 73.5%, 62.2%, 39.8%, and 11.6%, correspondingly. The median of systolic blood pressure and diastolic blood pressure was 135 (IQR_25%-75%_: 120–160) mmHg and 80 (IQR_25%-75%_: 75–95) mmHg, respectively. At presentation, 20 (7.9%) patients were in the Killip Class II or III and 19 (7.6%) patients had electrical rhythms other than the normal sinus rhythm. While 36 (14.3%) patients had ST-elevation in the anterior leads, 201 (80.1%) patients exhibited ST-elevation in the inferior leads.

Out of the 251 patients, cardiopulmonary resuscitation (CPR) was performed in 19 (7.6%) patients, intravenous inotrope was used in 14 (5.6%), and temporary pacemakers were implanted in 12 (4.8%) during their course of hospitalization. Ten (4.0%) patients died in the hospital, and 15 (6.0%) patients expired in the first month after myocardial infarction (MI). More detailed information about the course of hospitalization is provided in Table 3.

**Figure 3:**
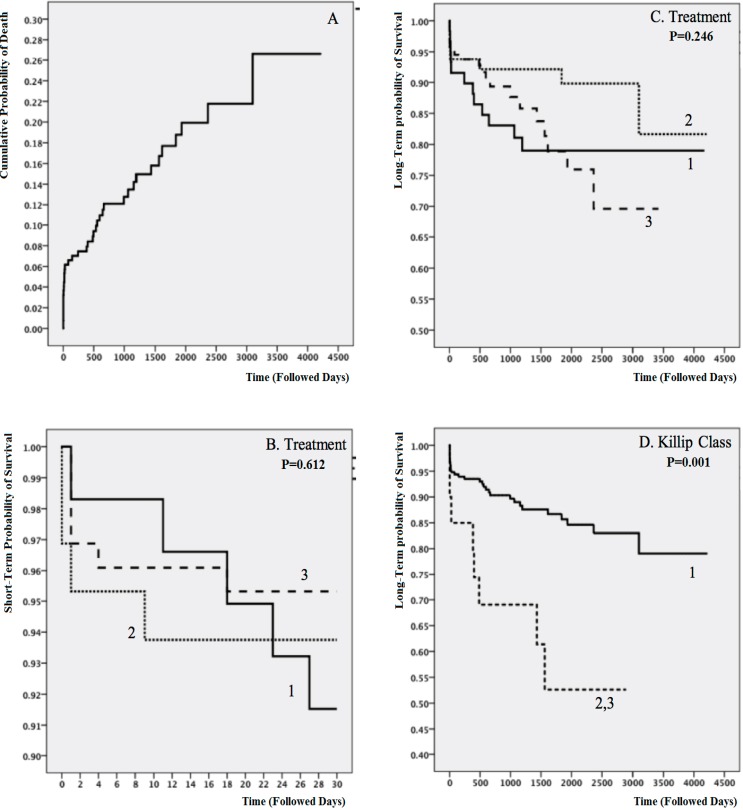
Cumulative hazard plot (A) and Kaplan–Meier graphs comparing survival between the study groups (B-D)

**Figure 4:**
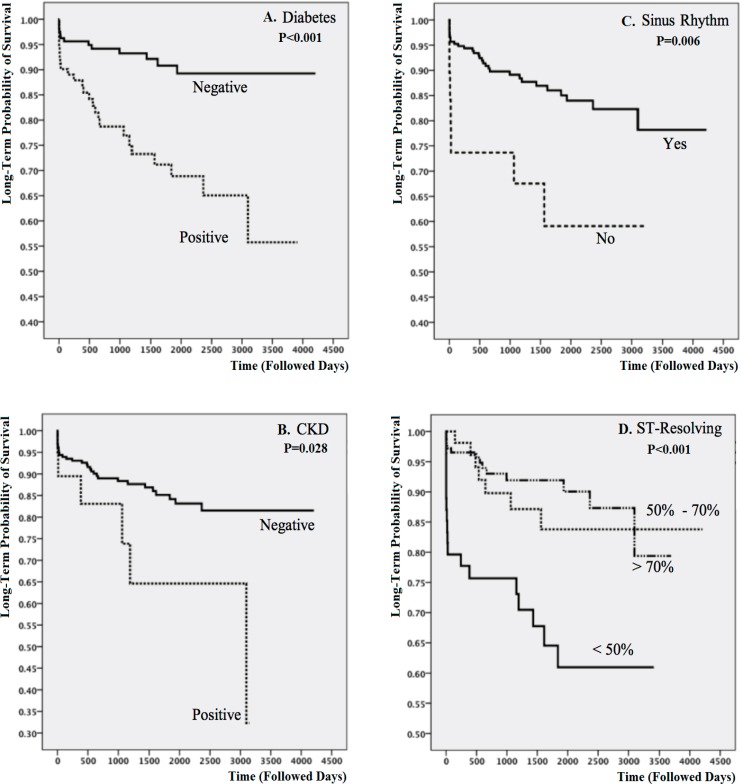
Kaplan–Meier graphs comparing long-term survival between the study groups (A-D)

The median time of follow-up was 1304 (IQR_25%-75%_: 571–2269) days, during which 38 (15.1%) patients died. During the follow-up period, 12 (20.3%) deaths were observed in the no-reperfusion group, 7 (11.0%) in the thrombolytic group, and 19 (15.2%) in the PPCI group (P=0.344). The survival analysis is depicted in Figure 3 and Figure 4. Despite showing some meaningful but statistically nonsignificant patterns, the 3 treatment strategies had no significant differences regarding both short- and long-term survival (P=0.612 for short-term survival and P=0.246 for long-term survival).

More detailed information regarding the initial treatment strategy and the next plan is given in Table 4. Among the 148 patients who underwent PCI finally, the culprit vessel was the saphenous vein graft in 78 (52.7%) patients and the native vessel in 70 (47.3%) patients, and both groups had similar survival (P=0.317 for short-term survival and P=0.525 for long-term survival). 

To find factors that contributed to the patients’ survival, we performed a univariate analysis first. (The results are presented in supplementary materials [Table S1, Table S2, and Table S3]). In the fully adjusted multivariate analysis (Table 5), undergoing CPR (HR: 15.06, 95% CI: 2.25– 101.14, P=0.005) was significantly associated with short-term mortality. In the long-term follow-up, having a history of diabetes (HR: 5.95, 95% CI: 2.03–17.44, P=0.001), opium abuse (HR: 4.85, 95% CI: 1.45–16.23, P=0.010), and undergoing CPR (HR: 11.73, 95% CI: 3.44–40.28, P=0.001) were significantly associated with increased all-cause mortality.

PPCI, Primary percutaneous coronary intervention

In plots B and C, 1 indicates no reperfusion, 2 indicates thrombolysis, and 3 indicates primary percutaneous coronary intervention.

B, short-term survival based on the treatment (P=0.246); C, long-term survival based on the treatment (P=0.612); and D, long-term survival based on the Killip Class on arrival (P=0.001)

A, survival based on diabetes (P<0.0001); B, survival based on chronic kidney disease (P=0.006); C, survival based on the presence of the sinus rhythm (P=0.006); and D, survival based on ST resolution (P=0.006)

**Table 1:** Baseline clinical status of the study population (n=251)*

	No Reperfusion (n=59)	Thrombolytic (n=64)	Primary PCI (n=128)	P
Baseline Characteristics				
Age (y)	65.5±9.77	61.9±8.62	64.3±9.59	0.254
Male	50 (84.7)	56 (87.5)	99 (77.3)	0.180
Interval from CABG (y)	6.0 (3.0-10.0)	7.5 (4.0-11.0)	8.0 (5.5-12.0)	0.071
Previous ejection fraction (%)	50.0 (45.0-55.0)	50.0 (45.0-55.0)	50.0 (45.0-50.0)	0.723
Cardiovascular Risk Factors				
Diabetes mellitus	24 (40.7)	18 (28.1)	49 (38.3)	0.278
Hypertension	42 (71.2)	39 (60.9)	104 (81.3)	0.009
Dyslipidemia	34 (57.6)	36 (56.3)	86 (67.2)	0.242
Smoking	24 (40.6)	32 (50.0)	44 (34.4)	0.271
Opium	9 (15.3)	9 (14.1)	10 (7.8)	0.223
Family history	17 (28.8)	23 (35.9)	41 (32.3)	0.700
Past Medical History				
PCI	6 (10.2)	4 (6.2)	20 (15.6)	0.032
CVA	3 (5.1)	0 (0.0)	2 (1.6)	0.116
CKD	7 (12.1)	6 (9.4)	6 (4.7)	0.175
COPD	1 (1.7)	1 (1.6)	0	0.349
Laboratory Data				
FBS mg/dL	107.1 (95.1-139.5)	105.4 (95.5-137.2)	111.8 (98.1-154.7)	0.182
HDL mg/dL	38.6 (31.6-46.2)	39.8 (32.7-46.1)	37.3 (33.5-46.0)	0.518
LDL mg/dL	98.2 (76.4-114.3)	98.6 (74.7-118.1)	86.9 (70.3-118.6)	0.114
TCH mg/dL	152.6 (137.1-182.3)	166.5 (133.2-189.6)	146.4 (125.1-186.6)	0.071
TG mg/dL	131.4 (87.1-165.3)	133.7 (96.4-180.1)	112.0 (83.4-157.7)	0.165
Hb g/dL	14.9 (13.2-16.1)	14.5 (13.5-16.2)	14.6 (12.9-15.7)	0.724
Cr mg/dL	1.10 (0.9-1.3)	1.00 (0.8-1.3)	0.95 (0.8-1.2)	0.289

CABG, Coronary artery bypass graft surgery; PCI, Percutaneous coronary intervention; CVA, Cerebrovascular accident; CKD, Chronic kidney disease; COPD, Chronic obstructive pulmonary disease; FBS, Fasting blood sugar; HDL, High-density lipoprotein; LDL, Low-density lipoprotein; TCH, Total cholesterol; TG, Triglyceride; Hb, Hemoglobin; Cr, Creatinine

* Data are presented as n (%), mean±SD or median (IQR_25%-75%_).

**Table 2:** Characteristics of the index myocardial infarction severity (n=251)*

	No Reperfusion (n=59)	Thrombolytic (n=64)	Primary PCI (n=128)	P
Clinical Examination				
Total ischemic time (hr)	-	3.0 (2.5-5.0)	4.0 (2.5-6.9)	<0.001
Systolic blood pressure (mmHg)	140 (120-160)	125 (111-150)	135 (125-160)	0.165
Diastolic blood pressure (mmHg)	85 (70-95)	80 (70-90)	85 (75-95)	0.159
Heart rate (bpm)	72 (64-89)	70 (56-82)	75 (65-86)	0.068
O_2_ saturation (%)	96 (95-96)	96 (95-96)	96 (95-97)	0.784
Killip Class II and III	9 (15.3)	1 (1.6)	10 (7.8)	0.020
First ECG Rhythm				
Normal sinus	51 (86.4)	62 (96.9)	119 (93.0)	0.087
AF	3 (5.1)	1 (1.6)	3 (2.3)	0.450
AV block	4 (6.8)	1 (1.6)	3 (2.3)	0.276
Paced	1 (1.7)	0	1 (0.8)	0.572
Junction	0	0	1 (0.8)	0.617
Location of ST-Elevation				
Anterior leads	5 (8.5)	10 (15.6)	21 (16.4)	0.336
Lateral leads	5 (8.5)	5 (7.8)	29 (22.7)	0.006
Inferior leads	51 (86.4)	55 (85.9)	95 (74.8)	0.077
Posterior leads	10 (16.9)	9 (14.1)	14 (10.9)	0.511
Right-side leads	14 (23.7)	21 (32.8)	49 (38.3)	0.145
Number of ST-elevation leads	4 (3-6)	5 (3-6)	5 (3-6)	0.560
Number of ST-depression leads	5 (3-7)	5 (4-7)	5 (3-7)	0.427
Number of leads with Q-wave	2 (2-3)	2 (1-3)	2 (1-3)	0.344
First troponin level (ng/L)	186.1 (29.3-740.1)	19.9 (8.5-91.4)	67.2 (19.6-314.7)	<0.001

PCI, Percutaneous coronary intervention; O_2_, Oxygen; ECG, Electrocardiogram; AF, Atrial fibrillation; AV, Atrioventricular block

* Data are presented as n (%) or median (IQR_25%-75%_).

**Table 3:** Course of hospitalization and the short-term outcome of the participants (n=251)*

	No Reperfusion (n=59)	Thrombolytic (n=64)	Primary PCI (n=128)	P
ST Resolution				
<50 %	24 (40.7)	10 (15.6)	20 (15.6)	<0.001
50–70 %	20 (33.9)	16 (25.0)	17 (13.3)	0.004
>70 %	15 (25.4)	38 (59.4)	91 (71.1)	<0.001
Course of Hospitalization				
Length of hospitalization (d)	8.0 (7.0-11.0)	7.0 (6.0-11.0)	6.0 (4.0-8.0)	<0.001
IV inotrope	4 (6.8)	3 (4.7)	7 (5.5)	0.878
Cardiopulmonary resuscitation	5 (8.5)	4 (6.3)	10 (7.8)	0.887
Endotracheal intubation	5 (8.5)	4 (6.3)	7 (5.5)	0.736
Ventricular tachycardia	3 (5.1)	0	3 (2.3)	0.182
Ventricular fibrillation	2 (3.4)	1 (1.6)	3 (2.3)	0.802
Electrical cardioversion	3 (5.1)	1 (1.6)	5 (3.9)	0.555
Intra-aortic balloon pump	0	1 (1.6)	0	0.231
Atrioventricular block**	6 (10.2)	2 (3.1)	5 (3.9)	0.138
TPM implantation	5 (8.5)	2 (3.1)	5 (3.9)	0.306
PPM implantation	0	0	0	-
Tamponade	0	1 (1.6)	0	0.231
Mechanical complication	0	0	0	-
Acute kidney injury	4 (6.8)	2 (3.1)	5 (3.9)	0.571
Major bleeding	0	1 (1.6)	3 (2.3)	0.493
Ejection fraction at discharge (%)	40 (35-45)	40 (35-45)	40 (35-45)	0.580
Outcome				
In-hospital mortality	3 (5.1)	3 (4.7)	4 (3.1)	0.772
30-day mortality	5 (8.5)	4 (6.3)	6 (4.7)	0.594

PCI, Percutaneous coronary intervention; IV, Intravenous; TPM, Temporary pacemaker; PPM, Permanent pacemaker

* Data are presented as n (%) or median (IQR_25%-75%_).

** Second degree or higher

**Table 4:** Details of the treatments performed (n=251)*

	1-Month Follow-up			Long-Term Follow-up		
	Died 15 (6.0)	Survived 236 (94.0)	P	Died 38 (15.1)	Survived 213 (84.9)	P
Reperfusion Strategy			0.610			0.344
No-reperfusion	5 (8.5)	54 (91.5)	-	12 (20.3)	47 (79.7)	-
Thrombolytic	4 (6.2)	60 (93.8)	-	7 (10.9)	57 (89.1)	-
Primary PCI	6 (4.7)	122 (95.3)	-	19 (14.8)	109 (85.2)	-
No-Reperfusion Subgroup			0.129			0.092
No CAG	3 (23.1)	10 (76.9)	-	5 (38.5)	8 (61.5)	-
Just CAG	1 (3.7)	26 (96.3)	-	6 (22.2)	21 (77.8)	-
CAG and PCI	1 (5.6)	17 (94.4)	-	1 (5.6)	17 (94.4)	-
Thrombolytic Subgroup**			0.179			0.208
No CAG	2 (22.2)	7 (77.8)	-	2 (22.2)	7 (77.8)	-
Just CAG	2 (6.3)	30 (93.7)	-	5 (15.6)	27 (84.4)	-
CAG and PCI	0	23 (100)	-	0	23 (100)	-
PCI Subgroup**						
IIb/IIIa inhibitor			0.737			0.413
Yes	2 (2.3)	88 (97.7)	-	11 (12.2)	79 (87.8)	-
No	3 (4.9)	58 (95.1)	-	39 (51.3)	37 (48.7)	-
Filtered wire			0.839			0.398
Yes	0	5 (100)	-	1 (20.0)	4 (80.0)	-
No	5 (3.5)	137 (96.5)	-	13 (9.2)	129 (92.8)	-
Thrombus aspiration			0.716			0.267
Yes	1 (3.2)	30 (96.8)		5 (16.1)	26 (83.9)	-
No	4 (3.4)	112 (96.6)	-	8 (6.9)	108 (93.1)	-
Target vessel			0.317			0.525
Native	4 (5.1)	74 (94.9)	-	7 (9.0)	71 (91.0)	-
SVG	1 (1.4)	69 (98.6)	-	7 (10.0)	63 (90.0)	-

PCI, Percutaneous coronary intervention; CAG, Coronary angiography; SVG, Saphenous vein graft

* Data are presented as n (%).

** Including both primary PCIs and delayed PCIs

**Table 5:** Multivariate analysis to find factors associated with short- and long-term survival (n=251)

	1-Month Mortality			Long-Term Mortality		
	HR	95% CI	P	HR	95% CI	P
Thrombolytic	2.71	(0.19 – 38.65)	0.462	1.14	(0.32 – 4.01)	0.837
Primary PCI	0.78	(0.08 – 7.21)	0.826	1.49	(0.55 – 4.03)	0.435
Previous EF	1.06	(0.88 – 1.28)	0.505	1.01	(0.93 – 1.09)	0.865
Diabetes mellitus	2.78	(0.60 – 12.89)	0.191	5.95	(2.03 – 17.44)	0.001
Hypertension	-		-	0.84	(0.28 – 2.48)	0.747
Opium	-		-	4.85	(1.45 – 16.23)	0.010
Smoking	-		-	0.83	(0.35 – 1.96)	0.671
PMH CVA	-		-	4.22	(0.69 – 25.90)	0.120
PMH CKD	-		-	1.94	(0.46 – 8.18)	0.366
FBS	-		-	0.99	(0.99 – 1.01)	0.493
LDL	-		-	1.01	(0.98 – 1.03)	0.915
TCH	-		-	0.99	(0.98 – 1.02)	0.894
Hb	-		-	1.04	(0.84 – 1.27)	0.739
Cr	1.47	(0.43 – 5.01)	0.537	1.58	(0.71 – 3.51)	0.262
Systolic blood pressure	1.01	(0.96 – 1.08)	0.646	-		-
Diastolic blood pressure	0.98	(0.88 – 1.09)	0.725	-		-
Heart rate	1.03	(0.98 – 1.08)	0.217	1.01	(0.99 – 1.04)	0.264
O_2_ saturation	0.96	(0.73 – 1.25)	0.756	-		-
Killip Class II-III	1.38	(0.04 – 52.50)	0.862	0.57	(0.17 – 1.91)	0.365
Normal sinus rhythm	0.12	(0.01 – 9.01)	0.334	0.39	(0.07 – 2.33)	0.305
AV block	25.44	(0.29 – 224.10)	0.157	1.62	(0.14 – 18.3)	0.695
Anterior MI	0.75	(0.11 – 5.06)	0.765	0.92	(0.28 – 3.03)	0.889
Number of STE leads	-		-	1.19	(0.95 – 1.49)	0.119
Number of Q-wave leads	1.01	(0.64 – 1.58)	0.967	0.91	(0.69 – 1.19)	0.487
First troponin level	1	(0.99 – 1.01)	0.694	-		-
ST resolution > 70%	1.63	(0.23 – 11.50)	0.624	0.52	(0.21 – 1.25)	0.144
Pulmonary edema	-		-	0.57	(0.09 – 3.43)	0.537
IV inotrope	9.30	(0.88 – 98.60)	0.064	3.84	(0.67 – 22.2)	0.132
CPR	15.06	(2.25 – 101.14)	0.005	11.73	(3.44 – 40.28)	0.001
TPM	3.60	(0.34 – 38.30)	0.288	2.44	(0.56 – 10.6)	0.236
AKI	1.65	(0.12 – 21.80)	0.702	0.89	(0.20 – 3.90)	0.875
EF at discharge	0.92	(0.76 – 1.12)	0.414	0.93	(0.85 – 1.02)	0.135

PCI, Percutaneous coronary intervention; EF, Ejection fraction; PMH, Past medical history; CVA, Cerebrovascular accident; CKD, Chronic kidney disease; FBS, Fasting blood sugar; LDL, Low-density lipoprotein; TCH, Total cholesterol; Hb, Hemoglobin; Cr, Creatinine; O_2_, Oxygen; AV, Atrioventricular; MI, Myocardial infarction; STE, ST elevation; IV, Intravenous; CPR, Cardiopulmonary resuscitation, TPM, Temporary pacemaker; AKI, Acute kidney injury

## Discussion

As of 2016, after the establishment of the 24/7 PPCI program in our center, all STEMI patients who had a timely hospital arrival (<24 h) or who arrived late but still had symptoms of ongoing ischemia underwent urgent coronary angiography (CAG), followed by PCI, if needed. Accordingly, all patients who received this treatment after 2016 were actually those who were initially treated at a different center before transfer to our hospital. However, before that, patients were treated based on the attending physician’s discretion. Of course, those who referred to us in the daytime had a higher chance of receiving PPCI than those who came during night hours.

Following the initial treatment, the decision as to whether or not to perform CAG was based on the physician’s clinical judgment. The patients who did not undergo CAG were evaluated via noninvasive tests; and in the absence of ischemic symptoms, CAG was not performed. After CAG, the next treatment strategy was at the physician’s discretion, considering the general condition of the patient, the symptoms, and the anatomy of the vessels.

In point of fact, the initial concept behind the design of the present study was to show that invasive treatment, even in patients with a history of CABG, would increase long-term survival. The results were, however, unexpected. Although we performed an adjusted multivariate analysis, it can be claimed that the patients in the medical treatment group had a better clinical condition. To establish the validity of this theory, we compared 3 different groups of patients with respect to factors that were potentially prognostic. The analysis showed that these different groups had comparable conditions and that the no-reperfusion group not only did not have a better clinical condition but was also worse off than the other 2 groups in terms of ST-segment resolution and diabetes.

Our study failed to demonstrate survival benefits from PPCI in this subgroup of patients; be that as it may, how can these results be justified? It should be noted that some of our patients in the no-reperfusion and thrombolytic groups were referred from other centers after initial management. Patients who died at the origin hospital before arrival at our center were not included in our study, which caused an underestimation of early mortality in these groups. 

PPCI in post-CABG patients differs in many ways from PPCI in other patients. Many reports, similar to our study, have shown a high rate of unsuccessful PCI,^4^^, ^^14^ an unsatisfactory final thrombolysis in myocardial infarction (TIMI) grade flow,^4^ and the inability to find the culprit vessel ^4^^-^^7^  in post-CABG patients. On the other hand, not only has the incidence of a TIMI grade flow 0/1 been reported less frequently,^7^ but also well-developed collateral vessels have been noted in this group of patients. Furthermore, more than half of the incidences of STEMI in normal patients are in the anterior territory, whereas anterior MI in patients with a history of CABG is much less prevalent.^4^^-^^7^ In half of the cases, the culprit is a vein graft,^4^^-^^7^ with several studies having shown the worse outcome of PCI on the vein graft in comparison with the native vessel.^4^^, ^^17^^, ^^18^  All the above mentioned points appear to detract from the value of invasive treatment, especially PPCI. Another point that should be considered in relation to the current investigation is that we evaluated and treated the entire study population in the best possible manner. Even those who did not undergo CAG were evaluated with noninvasive tests, and none of the patients was left unevaluated. This issue may have contributed to the similarity of the outcomes between our 3 study groups. 

The salient point in the interpretation of our results is that they, to some extent, run contrary to recently reported evidence elsewhere. Indeed, a recent large-scale study suggested that invasive management yielded similar outcomes in patients both with and without a history of CABG. This means that the benefits of invasive treatment in CABG-naïve patients can also be achieved in patients with a history of CABG.^7^

Our having considered all-cause mortality to be an end point has its own advantages and disadvantages. Two potential alternatives for measurement were major adverse cardiovascular events (MACE) and cardiac death, but both of them had some limitations. MI is an integral part of the definition of MACE; and because post-CABG patients usually have some degree of increased troponin levels, it is difficult to accurately determine the incidence of MI in their following admissions. Moreover, the fact that some patients may be admitted for MI in another center makes it virtually impossible to measure the true incidence of MACE. Since these patients have a high prevalence of comorbidities, there are also many limitations regarding the definition of a true cardiac death. It is also necessary to keep in mind that in a population with many comorbidities, a treatment that reduces cardiac death may not necessarily increase the overall survival. Although all-cause mortality has some degree of non-specificity, we finally resolved to consider it an end point.

The predictors of mortality have been studied thoroughly in the past, and numerous factors such as advanced age,^19^ lower systolic blood pressure,^19^^-^^21^ lower ejection fractions,^19^^-^^21^  advanced Killip classes,^   20 ^ indices of successful reperfusion,^21^ and renal failure^        22 ^ have been reported frequently. In the present study, our comprehensive univariate analysis found many contributing factors to the patients’ survival. In our fully adjusted multivariate model, however, due to the limited number of events, most factors lost their significance and a history of CPR during admission remained the sole independent predictor of short-term mortality. 

Our findings regarding long-term survival appear to be more interesting. Diabetes, opium abuse, and CPR during index admission were associated with long-term mortality in the multivariate-adjusted model. In recent years, more attention has been paid to the adverse effects of opium on the cardiovascular system. In a well-designed propensity score-matched study, opium abuse was found as an independent predictor of coronary artery disease and a dose-response relationship between opium abuse and the extent of coronary artery disease was reported.^3^ One year later, opium was introduced as an independent predictor of all-cause mortality, including cardiac death.^23^^-^^25^ Despite all the aforementioned evidence, unfortunately, there are traditional beliefs among Iranian patients and even many physicians in regard to the beneficial impact of opium. Intensive informative programs are required to raise awareness among patients and also physicians about the disadvantages and hazards of opium consumption.

The most prominent limitation of the present study is its observational design. Moreover, the fact that our study was not randomized may have caused selection bias. The small sample size is another salient weak point of our study, rendering it underpowered for the detection of the equality of these treatments.

## Conclusion

The method of treatment of STEMI patients with a history of CABG has undergone significant changes in recent years, shifting toward more invasive strategies such as PPCI. Our study failed to demonstrate the superiority of invasive treatment in terms of survival; however, our results should be interpreted in light of the fact that a small sample size and a limited number of events rendered our investigation underpowered. Far from expecting our results to be read as a measure of the inadequacy of PPCI, we wish to encourage further research on the advantages and disadvantages of this invasive modality in post-CABG patients.

Our results suggested that need for CPR during index hospitalization was an independent predictor of short-term mortality, whereas undergoing CPR during hospitalization, diabetes, and opium abuse were associated with lower long-term survival. 

## Notes:

***This paper should be cited as: ***Taghavi Shavazi M, Saadatagah S, Aghajani H, Poorhosseini H, Salarifar M, Amirzadegan A, Hajzeinali A, Alidoosti M, Aghajani R, Neamatipour E. Does Invasive Treatment Increase the Long-Term Survival of ST-Elevation Myocardial Infarction Patients with a History of Coronary Artery Bypass Graft Surgery? J Teh Univ Heart Ctr 2019;14(3):109-120.
